# Prioritisation and design of clinical trials

**DOI:** 10.1007/s10654-021-00761-5

**Published:** 2021-06-06

**Authors:** Anna Heath, M. G. Myriam Hunink, Eline Krijkamp, Petros Pechlivanoglou

**Affiliations:** 1 Child Health Evaluative Sciences, The Hospital for Sick Children, Toronto, ON Canada; 2 Division of Biostatistics, University of Toronto, Toronto, ON Canada; 3 Department of Statistical Science, University College London, London, UK; 4 Department of Epidemiology, Erasmus MC, University Medical Center, Rotterdam, Netherlands; 5 Department of Radiology, Erasmus MC, University Medical Center, Rotterdam, Netherlands; 6 Netherlands Institute for Health Sciences, Erasmus MC, University Medical Center, Rotterdam, Netherlands; 7 Center for Health Decision Science, Harvard T.H. Chan School of Public Health, Boston, MA USA; 8 Institute of Health Policy Management and Evaluation, University of Toronto, Toronto, ON Canada

**Keywords:** Type I and type II errors, Clinical trial design, Value-driven research, Research resources, Uncertainty, Value of information analysis

## Abstract

Clinical trials require participation of numerous patients, enormous research resources and substantial public funding. Time-consuming trials lead to delayed implementation of beneficial interventions and to reduced benefit to patients. This manuscript discusses two methods for the allocation of research resources and reviews a framework for prioritisation and design of clinical trials. The traditional error-driven approach of clinical trial design controls for type I and II errors. However, controlling for those statistical errors has limited relevance to policy makers. Therefore, this error-driven approach can be inefficient, waste research resources and lead to research with limited impact on daily practice. The novel value-driven approach assesses the currently available evidence and focuses on designing clinical trials that directly inform policy and treatment decisions. Estimating the net value of collecting further information, prior to undertaking a trial, informs a decision maker whether a clinical or health policy decision can be made with current information or if collection of extra evidence is justified. Additionally, estimating the net value of new information guides study design, data collection choices, and sample size estimation. The value-driven approach ensures the efficient use of research resources, reduces unnecessary burden to trial participants, and accelerates implementation of beneficial healthcare interventions.

## Introduction

Unnecessary or poorly designed clinical trials waste research resources [1] and delay implementation of effective interventions. A well-conducted trial can also waste resources if the collected information is irrelevant to patients, physicians, or healthcare policy makers. Importantly, wasted research resources and implementation delays negatively affect patients’ well-being and the efficiency of healthcare systems [1]. Therefore, before clinical trials are performed, we should assess and prioritise them based on their potential value and impact [2, 3].

In healthcare, research priorities can be set using several methods, e.g., using burden of disease or a qualitative assessment of the potential research impact [4]. These methods can identify some important research areas, but they do not use formal methodology to assess whether research is justified and how to design the best clinical trial. Therefore, they are unlikely to make the most efficient use of available resources [4–6].

Furthermore, trials are usually designed to control the type I and type II errors when making conclusions about the primary outcome of the trial using a statistical hypothesis test [7]. These primary outcomes are usually selected using expert consensus [5], rather than assessing the relevance of that outcome to clinical and policy decision making. Furthermore, the trial sample size, a key element of trial efficiency [8], is computed to control error rates [9]. This can lead to large sample sizes that cause feasibility issues, require excessive time and money, put an unnecessary burden on patients, and delay implementation of effective interventions [10, 11]. This paper defines this approach as the *error-driven* approach to clinical trial design.

Critiques of the *error-driven* approach highlight a risk of publishing misleading research findings [12] and a propensity to interpret research findings incorrectly [12, 13]. These errors mean that research efforts and resources are wasted and clinical and policy decisions are misinformed [13]. Therefore, to improve research impact and the use of research resources, we must approach clinical trial prioritisation and design differently.

Thus, this paper describes an alternative approach to research prioritisation and trial design that evaluates the *value* that research can provide to clinical and policy decision makers. This approach can help research groups prioritise and design future clinical trials that make the best use of limited research resources and ensure that trial evidence supports decisions around the use and reimbursement of interventions.

## Iterative research cycles: errors versus value

Healthcare research is an iterative process, where treatment effect estimates are contested or confirmed in successive studies. Throughout this process, researchers rarely claim certainty about a particular result and usually call for more research. Findings from trials add to the current evidence but uncertainty around the effectiveness or efficiency of interventions is rarely eliminated. However, clinicians and policy makers must make decisions, even in the face of uncertainty. Thus, we must determine if the remaining uncertainty justifies further research and how to design research that reduces uncertainty efficiently and effectively. Research should be an iterative cycle of designing studies, analysing the collected evidence in the context of what is known already, refining the research questions and designing future studies until we can make justifiable decisions about improving clinical practice.

### Error-driven approach

**Fig. 1:**
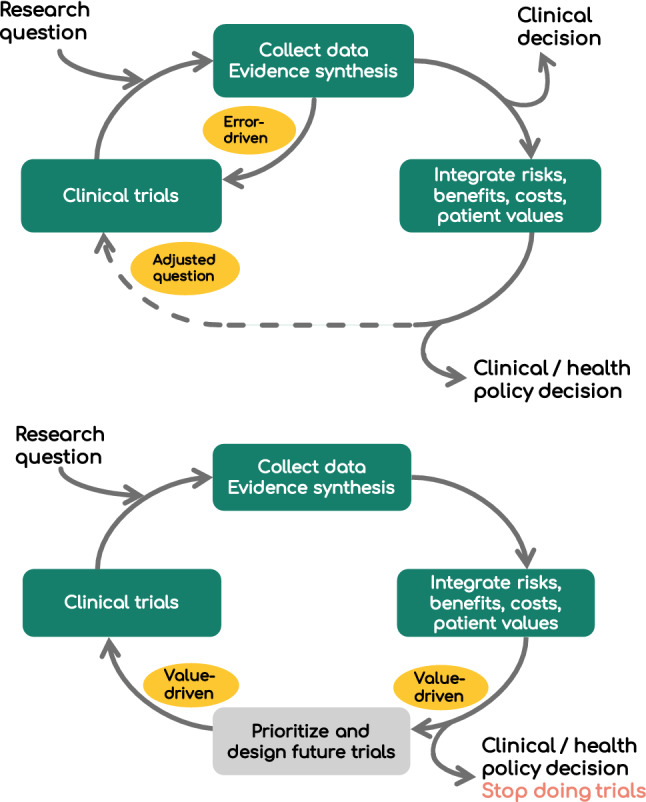
Iterative research cycles. **a** The current research cycle based on controlling type I and II errors. This classical method for developing and designing clinical trials is called the ‘error-driven’ approach. We consider that this approach has both a long and short iterative design process. The short route is in the top left-hand portion of the Figure and only iterates between the Evidence Synthesis and the Clinical trials boxes. The longer process includes all three key boxes while the dashed line represents the disconnect between how the information from the trials is used in policy making and the subsequent design of the next clinical trial. **b** A novel iterative research cycle that is driven by determining the value of different research strategies and pursuing research with the maximum value. This approach is called the ‘value-driven’ approach. Here the connection between policy making and the next clinical trial is determined using ‘value of information’ methods that prioritise and guide the design of future trials

Currently, the majority of clinical trials are designed using the *error-driven* approach [14]. All research starts with a research question (Fig. 1a). Using this research question, the design process proceeds with a systematic review of existing evidence from clinical trials, where feasible accompanied by a meta-analysis. Sometimes the results from the systematic review are combined with information on risks and benefits, patient values and costs to support clinical or health policy decision making [15]. These processes may be sufficient to guide decision making but lack of statistical significance for a treatment benefit in the synthesised evidence, coupled with evidence or an a-priori belief of a true benefit, often leads to a new clinical trial.

In the *error-driven* approach, trials are commonly designed by selecting a key outcome, known as the primary outcome, and fixing the trial sample size so that a statistically significant difference can be seen for this outcome when the trial results are analysed in isolation. Following this isolated analysis, the trial data may be added to the previous systematic review and meta-analysis and the research cycle starts anew.

In general, the primary outcome is selected using expert consensus [5], often considering feasibility, e.g., progression free survival is used in oncology trials to allow for shorter follow-up times. The sample size calculation is often based on currently available evidence about the baseline behavior of the primary outcome and expert specification of the “minimally important clinical difference” [16]. Using these two values, the sample size is set to control the type I error typically below a nominal level of 5% and the type II error below 10 or 20% [9]. Thus, the error rates, clinical judgement and an informal incorporation of existing information of treatment benefit are the key drivers of clinical trial design in the *error-driven* approach.

**Table:** 

Functions of “net value” used in health decision sciences and health technology assessment: $$Net \, health \, benefit \left( {NHB} \right) = HB - \frac{1}{WTP}*C$$ $$Net \, monetary \, benefit \left( {NMB} \right) = HB* WTP - C$$ Where: (1)HB is the health benefit, ideally integrating life expectancy and quality of life (e.g. QALYs) (2)C is the total costs, including healthcare and non-healthcare costs (3)WTP: society’s willingness-to-pay in monetary units for one unit of health *Example:* A treatment has an estimated health benefit of 12 QALYs (HB = 12), a cost of $200,000 (C = 200,000) and society’s willingness-to-pay is set to $50,000/QALY (WTP = 50,000). Then: $$NHB = 12 - \frac{1}{50,000}*200,000 = 8 QALYs$$ $$NMB = 12*50,000 - 200,000 = \$ 400,000$$	

Box 1.

### Value-driven approach

The *value-driven* approach asserts that research requires substantial investment of time, resources and money and should only be undertaken when it generates value. The value of research can be calculated using two key concepts: an estimate of the value of healthcare interventions and a suite of methods known as Value of Information (VOI) methods [17]. (For an explanation of the abbreviations used see Table 1).

**Table 1:** Table of abbreviations and definition used throughout the manuscript in alphabetic order and the associated units of measurement commonly used

Abbreviation	Full name	Units of measurement commonly used*
ENBS	Expected net benefit of sampling	Monetary units
EVPI	Expected value of perfect information	Monetary units
EVPPI	Population expected value of partial perfect information	Monetary units
EVSI	Expected value of sample information	Monetary units
Forgone benefit	Foregone benefit, or potential lost value, refers to the benefit that could have been gained if a more “optimal” decision had been made	Monetary units
HB	Health benefit	Health units, e.g. life years or QALYS
NBM	Net monetary benefit	Monetary units
NHB	Net health benefit	Health units, e.g. life years or QALYs
popEVPI	Population expected value of perfect information	Monetary units
popEVSI	Population Expected value of sample information	Monetary units
PSA	Probabilistic sensitivity analysis	not applicable
QALY	Quality adjusted life years	Is an unit of measurement that combines quality and the quantify of life years
RCT	Randomized controlled trial	not applicable
VOI	Value of information	not applicable
WTP	Willingness-to-pay	Monetary units per unit of health

*All value of information outcomes can alternatively be expressed in health units but this is less commonly done because it makes comparison with the costs of research more complicated

VOI methods are applicable irrespective of the method used to value the healthcare intervention. Nonetheless, we usually take a health policy making perspective and value interventions using a composite of health benefit and costs [17]. Health benefit is either measured using “hard” outcomes, such as the number of life years saved, or, more commonly, by combining quantity and quality of life into a measure known as quality-adjusted life years (QALYs) [18, 19]. Costs can include directly related healthcare costs alongside wider societal costs, such as productivity or leisure time loss [18]. Health benefits and costs are then combined into one of two composite outcomes: the net monetary benefit or net health benefit. Net health benefit (NHB) measures the number of health units saved by the interventions, while the net monetary benefit (NMB) is evaluated in monetary units, e.g. $ [20]. Both measures require an estimate of society’s willingness to pay (WTP) for one unit of health [21], which can be thought of as an “exchange rate” between health benefits and costs (Box 1).

Once the value of each intervention has been calculated, we can find the best intervention by considering which has the highest potential value. However, the available evidence on benefits and costs is uncertain, resulting in uncertainty about the intervention that maximizes value. Thus, VOI methods estimate the value of future research as the chance of making the wrong decision about the best intervention with the current level of evidence, multiplied by the benefit of changing the decision in settings where we would be wrong. Thus, the *value-driven* approach is based on understanding that a decision about the best intervention *must* be made following a trial and controlling the consequences and probability of incorrect decision making.

Figure 1b presents the *value-driven* approach, starting with a research question, data collection and evidence synthesis. The *value-driven* approach then estimates the value of each intervention using information and data on health outcomes and costs. This process uses methods from statistics, health economics and decision science to characterise the impact of uncertainty on the estimates of value. The current best intervention for use in clinical practice is the intervention that is expected to have the highest value [22]. VOI methods then formally assess whether the current evidence is sufficient to determine the best intervention [23].

To achieve this, we estimate whether the cost of undertaking additional research exceeds the value of the research (bottom Fig. 1b) [3]. We can also compute the value of alternative trial designs to prioritise the trial protocol with the greatest net value [24]. The *value-driven* approach then assumes that evidence collected in the trial will be analysed and interpreted alongside the current evidence to improve decision making following the trial. The value of each intervention can be estimated using the updated evidence and VOI methods can determine if further research is required. Thus, the *value-driven* approach is a full iterative research process.

## Steps of the value-driven approach

**Fig. 2:**
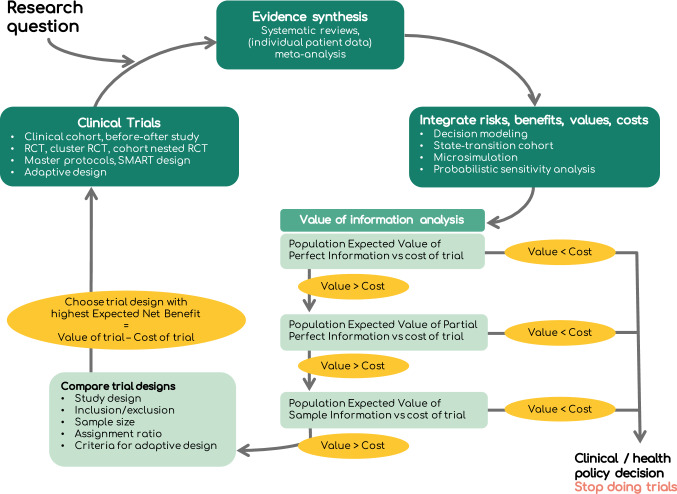
Details of the ‘value-driven’ approach. Table 2 gives explanations of each of the steps

The steps of the *value-driven* approach are summarised in Fig. 2 and Table 2. While these steps may seem cumbersome, recent methodological advances and software can facilitate the process [25–27]. We provide a clarifying example of the *value-driven* approach in Box 2.

**Table 2:** Steps of the value-driven approach to prioritise and design future clinical trials. The research question focuses on a decision problem in clinical medicine or public health. Each step uses specific methods. Interpretation of the results of each step affects whether it is useful to proceed to the next step

Specific question	Methods	Interpretation
What is already known?	Evidence synthesis, systematic reviews, (individual level) meta-analysis	Review and calculate summary measures of the best-available evidence
What is best for our patient population?	Decision modeling, state-transition cohort model, microsimulation	Expected value of each strategy is the value that we can expect on average given the current best-available evidence on risks, benefits, costs and patient values
How certain are we that the decision is the best choice?	Probabilistic sensitivity analysis (PSA)	Propagate the uncertainty in the input parameters through the model to estimate the uncertainty in the outputs
What is the value of eliminating all uncertainty around all parameters, per patient?	Expected value of perfect information (EVPI)	The expected value for the hypothetical scenario where further research eliminates all decision uncertainty, meaning that we would know the exact values of all parameters
What is the value of eliminating all uncertainty around all parameters, considering all patients that could benefit?	Population expected value of perfect information (popEVPI)	popEVPI equals the EVPI per patient multiplied by the number of patients that can benefit from the new information. The popEVPI is the maximum obtainable value of performing more research. It needs to exceed the cost of performing new research in order to proceed to the next step. This is a necessary but not sufficient condition to do more research
Which parameters are driving the uncertainty around the decision?	Expected value of partial perfect information (EVPPI)	EVPPI calculates the EVPI of a limited subset of parameters. The EVPPI guides the design of a future study by focusing the data collection on those parameters that are the most valuable to collect
What is the value of reducing uncertainty rather than eliminating it, per patient?	Expected value of sample information (EVSI)	EVSI is the expected value of reduction in uncertainty by collection of a limited subset of parameters in a study with limited sample size, which depends on the sample size of the envisioned trial
What is the value of reducing uncertainty, considering all patients that could benefit?	Population expected value of sample information (popEVSI)	popEVSI is the EVSI per patient multiplied by the number of patients that can benefit from the new information
Determine the ‘’cost’’ of doing more research	Resources for new study + foregone benefit	Cost of a new study includes the resources required to perform the study, the foregone benefit due to delayed implementation of a potentially beneficial intervention, and the foregone benefit due to allocation to a suboptimal strategy in an RCT
Does the information provided by a new trial justify its cost?	Expected net benefit of sampling (ENBS): popEVSI of the trial – Cost of the trial	ENBS is the expected net benefit of reducing uncertainty by collection of data depending on sample size of the trial. ENBS needs to exceed zero in order to justify performing the trial
Which trial design is optimal?	Compare the ENBS of different trial protocols	Choose the trial design that maximises the ENBS. Choices are the study design, inclusion/exclusion criteria, sample size, assignment ratio, criteria for adaptive design

Firstly, the *value-driven* approach determines the clinical or public health decision making problem that is relevant to the research question. Next, we summarise the available evidence using systematic reviews and meta-analyses [28], integrate evidence on benefits and costs using a decision model [15] and calculate the *expected value* of each intervention.

Following this, we use distributions around the input parameters to model uncertainty in the current evidence and propagate this uncertainty through the decision model using “probabilistic sensitivity analysis” (PSA) [15]. PSA determines the effect of input parameter uncertainty on the expected NMB or NHB. Using the PSA results, VOI methods can determine the chance and the consequences of making the wrong decision about the best treatment, i.e., the value of future research (Fig. 2 and Table 2). Strictly speaking, VOI is the “expected cost of the uncertainty” where ‘’cost’’ is expressed in foregone health benefit or monetary units. Foregone benefit, or potential lost value, refers to the benefit that could have been gained if a more “optimal” decision had been made.

A VOI analysis begins by calculating the value of eliminating all sources of parameter uncertainty, known as the expected value of perfect information (EVPI). EVPI is the upper limit on the value that can be generated from a future study collecting evidence about the model parameters. If the EVPI is low, then no future research should be proposed [29].

As a clinical trial is unlikely to estimate all parameters that are relevant to the decision, a VOI analysis proceeds by considering which outcomes should be included in the future study by identifying the parameters that would generate the most value if we were to gather more information about them. This is assessed by computing the value of eliminating uncertainty in a smaller group of parameters using the expected value of partial perfect information (EVPPI) [30]. EVPPI is computed for different groups of parameters and those with the highest value should be considered as study outcomes. This information about the study outcomes of interest, helps to select the most appropriate study design. For example, a VOI analysis can help us to answer the question: ‘’should we undertake a longitudinal cohort study to determine incidence or a randomised controlled trial (RCT) to determine treatment effect?”.

The final VOI design phase uses the expected value of sample information (EVSI) to determine whether a specific trial design would give value. To undertake this final analysis, EVSI must be scaled up by the number of patients who could benefit from the research results to compute the population EVSI (popEVSI). If the popEVSI exceeds the cost of the proposed trial, the trial has value. To facilitate this analysis, we compute the expected *net* benefit of sampling (ENBS), defined as the difference between the popEVSI and the study cost [3]. If ENBS is less than zero, then this trial is not valuable, and the current best intervention should be used in clinical practice. Finally, ENBS can be computed for different study protocols by changing the study type, inclusion/exclusion criteria and sample size, to determine the most valuable trial [24]. Given that there is a cost associated with enrolling participants, the *value-driven* approach enrols participants until the value of their data is smaller than the cost of enrolling them in the trial [24].

## How the value-driven approach addresses challenges in trial design

This section outlines how the *value-driven* approach can offer solutions to challenges researchers have to deal with when developing and performing clinical trials.

### Efficiency

Clinical trials are expensive and time-consuming, mainly related to the required number of trial participants. The *value-driven* approach assumes that trial data is analysed within the totality of evidence relevant to the policy decision. This can reduce the sample size, cost and patient burden.

The *value-driven* approach also improves trial design efficiency when multiple interventions are available. In the value-driven approach, decisions about which interventions to include are made by considering which interventions are key to determining the best intervention upon completion of the research process. The *error-driven* approach uses expert consensus to determine the trial interventions, which may exclude valuable interventions from the trial.

Finally, the time required for research and reimbursement decisions delays implementation, which can have health consequences as effective treatments are slow to reach patients (see Box 2). The *value-driven* approach addresses the foregone benefits of delayed implementation explicitly as a cost of further research. Furthermore, it ensures that information for decision/policy making is available at the start of the trial and can be updated following the trial. Finally, clinical trial design is optimised to support decision making. The downside of this approach is that in-depth analysis is required in the trial planning phase, which requires more time and resources.

### Generalisability

Clinical trials are often criticised for their lack of generalisability [31] and their use of outcomes with limited relevance to clinicians, patients or policy makers [32]. The *value-driven* approach addresses these issues by ensuring that trial outcomes will support decision-making. The value of trials collecting evidence on alternative outcomes, i.e., short-term surrogate outcomes vs long-term outcomes, can be compared to their required resources. This would determine whether the additional information in the long-term outcomes is worth the increased complexity and cost. The value of a trial is also proportional to the number of people affected by the decision (Table 2). Thus, if the trial has limited generalisability, the value of the trial is limited. This supports the development of trials with wide inclusion criteria. Moreover, reducing the time between trials and their implementation ensures that trial information more closely reflects current practice.

**Table:** 

**Example of Value-driven approach in trial design** Willan and Kowgier compared value of information methods to traditional power calculations [33]. Example: A randomized clinical trial (RCT) funded by the Canadian Institute of Health Research (CIHR) investigating early vs late external cephalic version (ECV) for pregnant women presenting with a fetus in breech position. Error-Driven Approach: Primary outcome: Non-Caesarean delivery Sample Size Calculation: The investigators of the trial used evidence from a pilot study (﻿n = 116 in both arms, where the proportion of non-Caesarean deliveries in the early ECV arm was 35.3% compared to 28.4% in the late ECV arm) [34]. The minimally clinically important difference was determined to be an 8-percentage-point increased probability of a non-Caesarean delivery in the early ECV arm. The type-II error rate of the trial was set to 0.20 with a two-sided type-I error of 0.05. Thus, the trial had an 80% probability of correctly rejecting the null hypothesis if the treatments differed by eight percentage points or more and a 5% probability of incorrectly rejecting the null hypothesis if there was no difference between treatments. The sample size was calculated for a two-sample test for proportions, including a continuity correction to adjust for binary outcomes [35]. Sample Size: 730 patients per arm This large trial was successfully funded by CIHR and completed in 2008 [36] Value-Driven Approach: Estimating Value: The prior distribution of the incremental net benefit was estimated based on the pilot data (probability difference (41/116 – 33/116)) combined with the assumed ﻿societal willingness-to-pay of $1,000 to achieve a non-Caesarean delivery. To estimate the total number of patients affected by the decision, a time horizon of 20 years and annual North American incidence of 100,000 breech presentations was assumed Sample Size Calculation: A decision model was developed to estimate the effects of both strategies based on the published pilot data. Next the uncertainty around the decision was simulated and the expected value that can be gained by reducing uncertainty (EVPPI) was calculated. This expected value of new evidence minus the cost of collecting this information yielded the expected net benefit of further research (ENBS). The sample size that maximizes the ENBS was selected as the optimal sample size.﻿ Sample Size: 345 patients per arm The value-based approach would have resulted in a 52.7% reduction in trial sample size *Efficiency*: The required trial budget would be reduced from $2,836,000 to $1,604,000 (43.4% reduction) The expected net monetary benefit of the trial would increase from $179,000 to $736,383 (around 4 times higher) Note that the two approaches take different perspectives allowing the value-based approach to require a lower sample size than the 730 patients required to achieve statistical significance in the error-driven approach. The value-based approach does not aim to achieve statistical significance. Instead, it optimizes the trade-off between collecting more information, which is costly, and making an incorrect decision about the best treatment. The value-based approach considers that a decision can be made between two treatments, even if the difference between them on some clinically-relevant outcome is not statistically significant [22].This allows the value-driven approach to potentially increase trial efficiency to such an extent that justifies the added complexity of designing research with this approach [33]. Note that Willan and Kowgier [33] also consider a two-stage value-based approach which increases the expected net benefit of the trial still further to approximately 8 times higher than that of the error-driven approach.	

Box 2.

### Validity

Clinical trials can have issues with validity as patients switch interventions, are lost to follow-up and do not follow protocol [1, 37]. While treatment switching and protocol adherence are issues for the *value-driven* approach too, we can assess the impact of losing patients to follow-up and we can value efforts to reduce loss-to-follow up, i.e., financial incentives for follow-up questionnaires [38]. Furthermore, trial simulations that consider these issues can define how they influence the value of the trial.

### Feasibility

The *value-driven* approach directly considers the available budget, the time-taken to undertake the trial and the delay in widespread implementation [3]. Thus, the *value-driven* approach designs trials that are, by definition, feasible conditional on budgetary and time constraints. Conversely, the *error-driven* approach can lead to designs that are infeasible, i.e., requiring infeasible sample sizes in rare diseases [39].

### Personalised and precision medicine

Precision medicine is becoming an important part of healthcare [40, 41] but causes methodological issues in trial design [42]. However, by focusing on supporting personalised decision-making, the *value-driven* method can offer alternative trial designs that are feasible and generalisable. Furthermore, novel *value-driven* methods are available to optimise the design of trials in precision medicine [43].

### Emerging technologies

Finally, fast evolving technologies can mean that interventions are outdated before trial completion. The *value-driven* approach considers that trial evidence will be added to the current evidence, facilitating adaptive trials compared to the *error-driven* approach. We can assess the value of new interventions and compute the value of adapting the trial to include them. Thus, the *value-driven* approach includes flexibility that ensures evidence is relevant to decision-making, even in the face of emerging technologies and a changing research landscape.

## Discussion

This paper proposes the *value-driven* approach as an alternative to the current *error-driven* approach for clinical research studies, focusing on clinical trial design although these methods are also applicable to observational studies. We now discuss project- and system-level barriers to the widespread implementation of the *value-driven* approach and highlight the potential benefits of the approach.

### Time required for Trial Design

Under the *value-driven* approach, it takes more information and time to design a trial as the value of each intervention must be estimated using decision modelling. This requires, ideally patient-level, evidence on the interventions’ costs and benefits as well as their effect sizes. In contrast, the *error-driven* approach focuses on a key primary outcome for interventions that have been selected for the trial using expert consensus. Thus, the *value-driven* approach requires a wider literature search and different modelling and data synthesis methods. However, these analyses are required for policy making and thus, we can reduce the time for trial data analysis by including them at the design stage. The increased time and cost of the research prioritisation and trial design process will require additional funds. However, as the *value-driven* approach optimises the spending of research resources, the savings from efficient and effective trials are expected to recoup the cost of this design phase.

### Data access

The decision modelling required in the *value-driven* approach and the final trial analysis should include all the currently available data. This includes data in aggregated form from the literature and patient-level data from previous trials. Accessing these data to design a new trial may be challenging and, if these data are not made available, then the *value-driven* approach could develop designs that result in inefficient use of resources. However, wider data access and the secondary use of trial data are increasingly used to improve the efficiency of healthcare research [44]. In addition, recent efforts by academic journals on data sharing (e.g. Plos ONE requirements for public data access[45]) can facilitate such data access, and further efforts by other academic journals would facilitate this. Thus, reusing data to improve trial design and to ensure that research effectively targets decision uncertainty should be a key part of this effort.

### Expertise required for Trial Design

The *value-driven* approach requires collaboration with an interdisciplinary group of researchers, including trialists, statisticians, health economists, and decision modelers for the trial design. There is a lack of expertise among trialists and statisticians with VOI methods, in part because the methods have been challenging to implement [26]. However, recent research has focused on facilitating VOI analyses [26, 46, 47] reducing the barriers to implementation by increasing education and software [25, 48–51]. Including researchers familiar with cost-effectiveness and VOI methods in the trial design will increase costs, again, offset by the more seamless and efficient use of resources in the trial and its analysis. Specifically, this collaboration ensures that information required for cost effectiveness analyses can be collected in the trial outcomes.

### Adaptive research questions

If funding is available for the design and conduct of a trial, challenges may arise if the VOI analysis indicates that the proposed trial is an inefficient use of research funding, e.g., an alternative study or a smaller sample size may be required. In this case, funding may need to be returned or repurposed. Flexible funding instruments would allow researchers to undertake valuable research, even when it was not originally proposed. To benefit fully from the *value-driven* approach, the current method of research funding where deliverables are pre-specified will require modification.

### Status Quo in regulatory processes

Regulatory authorities worldwide have strict guidelines around the type of evidence that must be submitted to demonstrate treatment efficacy and safety. If a trial is developed as the basis of a submission to these regulatory bodies, innovative trial designs, such as those developed through the value-driven approach, may be limited by those guidelines. However, there is an increasing trend for regulatory authorities to become more flexible and acceptive of innovative and efficient trial designs (e.g., umbrella/basket trial designs) given the challenges facing the current regulatory landscape (e.g., personalized medicine, expedited access). Additionally, value-driven approaches can suggest expanding the data collection beyond the typical primary efficacy/safety outcomes (e.g., costs or quality of life), thereby strengthening the evidence submitted to a regulatory body.

### Current research infrastructure

The current research infrastructure and publication culture support the *error-driven* approach, e.g., statistically significant results increase the chances of publication in high impact journals [52], and analysing trial results within a decision model is less well accepted. However, the *error-driven* approach has been heavily criticised [13] and journals are beginning to accept trials analysed using alternative methods [53].

In conclusion, the *value-driven* approach has advantages over the *error-driven* approach as research performed based on a *value-driven* trial design will collect data that are valuable to society and thus reduce research waste [54]. The *value-driven* approach can also justify a more streamlined implementation of interventions, which is particularly important when facing an urgent situation affecting a large number of patients. The *value-driven* approach can guide the choice of study type, inclusion/exclusion criteria, sample size, allocation ratio, and criteria for adaptive designs.
